# Dynamic colour change predicts movement behaviour in a diadromous fish

**DOI:** 10.1111/jfb.70331

**Published:** 2026-02-19

**Authors:** Joshua S. Barrow, John R. Morrongiello

**Affiliations:** ^1^ Biosciences 4, School of Biosciences The University of Melbourne Parkville Victoria Australia

**Keywords:** adaptive colouration, camouflage, crypsis, diadromous, dynamic colour change, fish behaviour, movement, predation risk, visual ecology

## Abstract

Dynamic changes in colour and pattern facilitate key behavioural functions in animals, particularly camouflage for predator avoidance. However, the benefits of colour change depend on the environmental and behavioural contexts. We tested how colour change interacts with movement behaviour in a freshwater fish by filming individuals in an open‐field test following a stressful stimulus. Fish with lighter patterns, brighter colour and/or those that changed to lighter colours were more likely to move. Our results highlight the interconnected roles of behaviour, colour change and environmental context in effective camouflage.

Many animals are capable of dynamically changing their colouration and patterning, a trait that enables a range of important ecological and behavioural functions (Duarte et al., 2025). Colour change can occur via two broad mechanisms: physiological colour change, which involves rapid reversible changes in pigment distribution occurring over seconds to minutes, and morphological colour change, which involves slower changes in pigment production, tissue structure or moulting and typically occurs over days to seasons (Figon & Casas, 2018). These mechanisms are distributed widely across the animal kingdom, from beetles to bearded dragons and prawns (Dickerson et al., 2020; Duarte et al., 2025; Green et al., 2019; Umbers et al., 2014; Zimova et al., 2018).

Some of the most iconic colour‐changing animals display colour change alongside other key behaviours. For example, chameleons flash bright colours during aggressive displays towards rivals via physiological mechanisms (Ligon & McGraw, 2013), while snowshoe hares undergo seasonal morphological colour change to match changes in habitat (Zimova et al., 2018). In contrast, cuttlefish shift their skin patterns almost instantaneously to blend into their surroundings and hide from predators or prey through highly controlled physiological colour change (Chiao et al., 2015). Together, these examples illustrate that colour and pattern are not fixed traits: they are dynamic responses operating over distinct timescales and are often coordinated with other behaviours (Duarte et al., 2017).

Animals such as fish, amphibians and reptiles change colour by actively controlling specialised skin cells known as chromatophores (Stuart‐Fox & Moussalli, 2011). These include melanophores (containing dark melanin pigments), xanthophores and erythrophores (yellow to red pigments), iridophores (structural reflectors that produce iridescent or blue–green colours) and leucophores (light‐scattering cells that appear white) (Fujii, 2000). Together, these chromatophore types enable colour change through both the redistribution of pigments and through changes in the molecular architecture and spatial arrangement of structural elements within them (Figon & Casas, 2018). In freshwater fish, for instance, melanophores are a key driver of colour change: when melanin is aggregated at the centre of the cell, the skin appears light, whereas when melanin is dispersed throughout the cell, the animal appears dark (Sugimoto, 2002).

For many freshwater fish inhabiting relatively shallow habitat, camouflage plays a critical role in preventing predation, particularly from birds (Whitaker et al., 2021). These animals often undergo rapid colour changes to match complex backgrounds and remain undetected. While stationary, fish might display complex, high‐contrast, patterning that breaks up the body outline and reduces the likelihood of being detected (Drerup et al., 2024). However, fish need to move to feed, explore new habitat and reproduce (Cooke et al., 2022). Maintaining a high‐contrast colour pattern while moving may be disadvantageous and risky in some contexts as fish traverse through a heterogeneous habitat with potentially increased encounters with predators (Furey et al., 2016). In other contexts, high‐contrast patterns might be favoured when a fish is moving because it can become blurred to a predator, enabling better background matching and reducing detection. This perceptual phenomenon is known as the flicker fusion effect (Umeton et al., 2017).

The aquatic light environment through which fish move is itself heterogenous due to streambed features, instream vegetation, riparian shading and caustic patterns formed by the light refraction through surface waves (Cuthill et al., 2019). Camouflage can be enhanced when moving through a dynamic light environment by changing to a lighter, more uniform and lower‐contrast pattern, or in some cases a higher‐contrast pattern. However, low‐contrast colouration is thought to mimic the shifting light environment created by caustics, causing moving objects to blend into the background and thus making them harder to be perceived by predators (Zylinski et al., 2009). Ultimately, the benefits of colour change are context dependent, where a colour shift might be advantageous in one situation, but disadvantageous in another.

We tested the hypothesis that moving and sedentary animals would have different colour patterns and propensity for colour change by performing behavioural assays on a freshwater fish. We aimed to determine whether a fish's colour pattern and the direction of any colour change determined their propensity for movement following exposure to a stressful stimulus in a novel environment.

Tupong (*Pseudaphritis urvilli*) are a diadromous fish native to southeastern Australia. Tupong express a range of colours and patterns depending on the habitat in which they live, including browns, blues and reds, with combinations of banding patterns and lines (Bray & Thompson, 2024). We collected 36 tupong using backpack electrofishing (Smith‐Root model LR‐20B) from four coastal rivers on Wurundjeri, Bururong and Gunaikurnai country in Victoria Australia: the Bunyip and Tarago Rivers, Eumermmering Creek and Cardinia Creek. Following capture, fish were placed in fresh river water in a large 50 L fish bin for a 45–60 min recovery period. Once the recovery period concluded each fish undertook an open field test to assess their propensity for movement. Experiments were conducted on the bank of the river from which the fish were collected. Fish were placed separately into white 12.5 L experimental containers that were covered with a black lid and left for 5 min to acclimate. The lid was then quickly removed and the fish exposed to bright sunlight. This rapid light change provided an assumed stressful stimulus that was expected to induce a behavioural response and colour change in the fish. Each fish was filmed for 10 min using a GoPro 5 attached to a tripod. We repeated the trial three times for each fish, separated by a 30 min recovery period, where fish were moved into individual 10 L clear containers in fresh river water and placed in the shade. Fish were returned to the stream of capture after the final trail.

We manually analysed the 108 10 min trial videos, noting whether fish moved (binary response) throughout each trial. We then categorised the colour pattern that each fish expressed for the longest period of time (dominant colour pattern) during each trial (one of three levels, see results) and recorded whether it got darker, lighter or did not change its colouration throughout the trial. Fish were only ever observed changing colour pattern once during a trial (i.e. no fish observed changing multiple times in a trial), which typically occurred immediately after stimulus. Finally, we took a frame capture from a video of each fish while it was displaying its dominant colour pattern and used Fiji image analysis software (Schindelin et al., 2012) to quantify total body brightness (measured in pixel intensity). Due to localised differences in cloud cover and tree shading, brightness values were not directly comparable among individuals. We therefore standardised our brightness measurements by dividing the mean brightness value for each fish by the mean brightness value of its white tub background (Morrongiello et al., 2010). We then accounted for the potential influence of location‐specific differences in water clarity and colour affecting the expression of fish colour. This was done by scaling each fish's brightness value relative to other individuals collected at the same time and location. Here, individual brightness scores were standardised within each sampling location by converting values to *z*‐scores (subtracting the location‐specific mean and dividing by the location‐specific standard deviation). More positive values represented relatively lighter fish and more negative values represented relatively darker fish.

We calculated the repeatability score (*R*) for movement and for colour change across each fish's three exploratory behaviour trials using a Bayesian generalised linear mixed model in the brms package in R (Bürkner, 2017; R Core Team, 2024). To determine the most appropriate repeatability structure, we developed models with (1) a random intercept for fish identify, (2) a fixed effects of trial plus a random intercept for fish identity and (3) a fixed effects of trial plus random slopes of trial within fish identity. Models were compared using leave‐one‐out cross‐validation, which estimates out‐of‐sample predictive accuracy for Bayesian models while accounting for model complexity and avoiding overfitting. The best performing model in the movement model comparison included a fixed effect of trial plus random slopes for trial within fish identity, and the best performing model from the colour change model comparison included just a random intercept for fish identity. These model structures were then used to calculate the repeatability scores. Repeatability was calculated as the proportion of total variance attributable to differences among individuals ($R=\sigma_{\mathrm{ind}}^{2}/\sigma_{\text{total}}^{2})$ and 95% credible intervals were obtained from the posterior distributions of variance parameters.

We found that an individual's propensity to move across trials was moderately repeatable [*R* = 0.59, confidence interval (CI) = 0.007–0.93], as was their propensity to change colour (*R*_lighter_ = 0.43, CI_lighter_ = 0.002–0.85; *R*_darker_ = 0.86, CI_darker_ = 0.63–0.95). Next, we created three separate generalised linear mixed models (GLMMs) with binary error distributions to determine whether movement (0, 1) was linked with tupong colour, colour change or brightness. A random intercept for each fish accounted for the repeated measures nature of the data. All colouration models were compared against an intercept‐only null model using AICc.

Tupong displayed three distinct colour patterns during our study: crossways dark banding (including very dark body colour with banding), dark lengthways stripes on flanks and light brown all over (see examples in Figures 1 and S1). Across all trials (including repeated trials from the same individuals), dark banding was the predominant colour pattern in 51.85% of trials, dark stripes in 30.55% of trials and uniform light brown in 17.60% of trials. We found that colouration was dynamic, with fish often rapidly changing patterns. Overall, 24.07% of trials (including repeat trials) had fish that darkened their colour, 11.11% had fish that lightened their colour and 64.81% had fish that showed no colour difference throughout a trial (see Figure 1 for examples).

**FIGURE 1:**
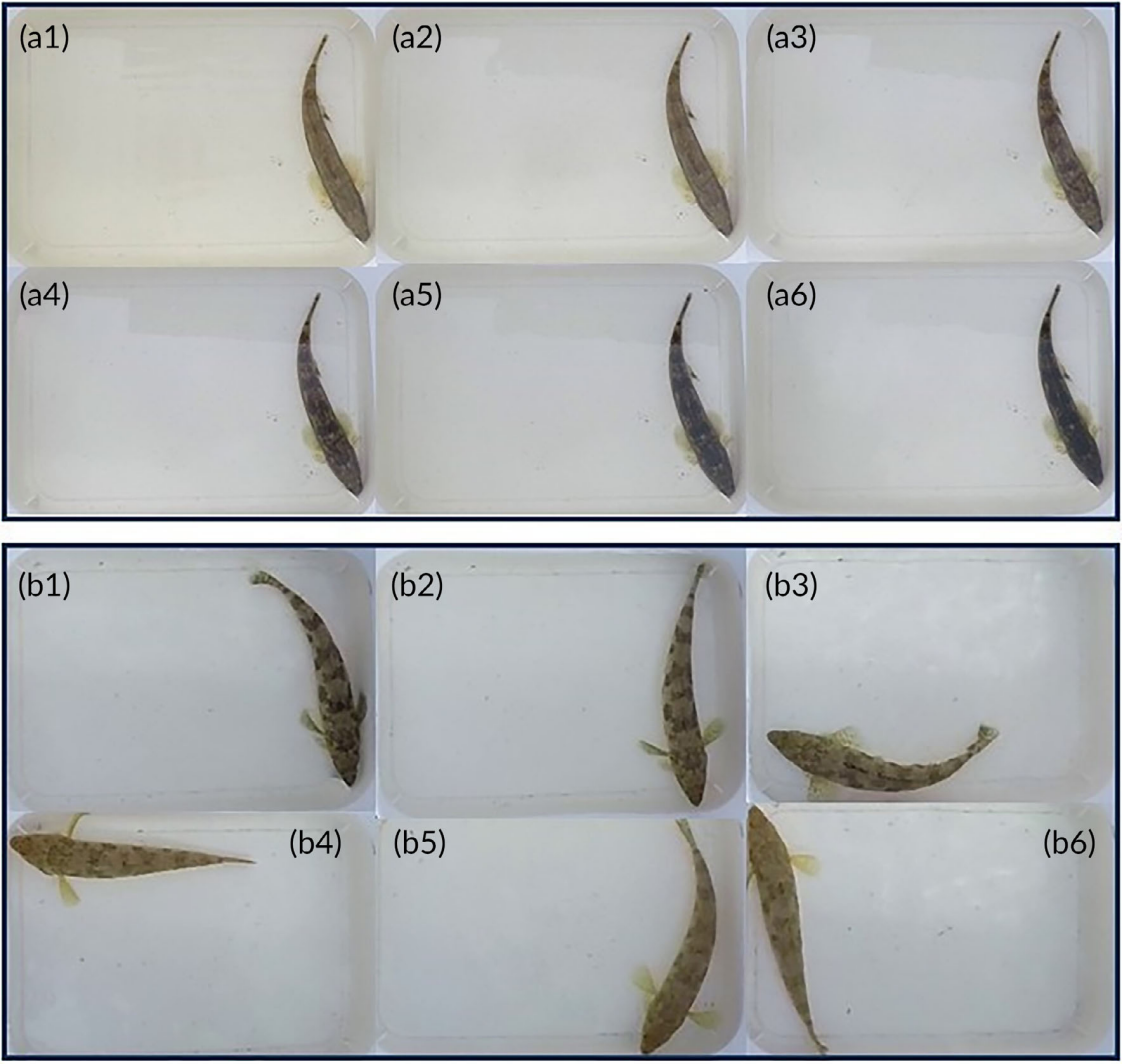
Sequential images from behavioural trials of two tupong (A and B). Images show how colouration changes at six time points in each trial (numbered 1–6). Fish (A) became progressively darker and fish (B) progressively lighter. Fish (A) displayed no movement, while fish (B) moved extensively around the container.

In all three model comparisons, we found clear evidence that colouration could predict the probability of movement (Table S1). Individuals had a significantly greater probability of moving if they had lighter colour pattern [regression coefficient *β* = 4.031, standard error (SE) = 1.331, *p* = 0.002] (Figure 2a), higher brightness values (*β* = 0.733, SE = 0.348, *p* = 0.035) (Figure 2b) or if they changed their colour to be lighter (*β* = 21.084, SE = 7.152, *p* = 0.003) (Figure 2c).

**FIGURE 2:**
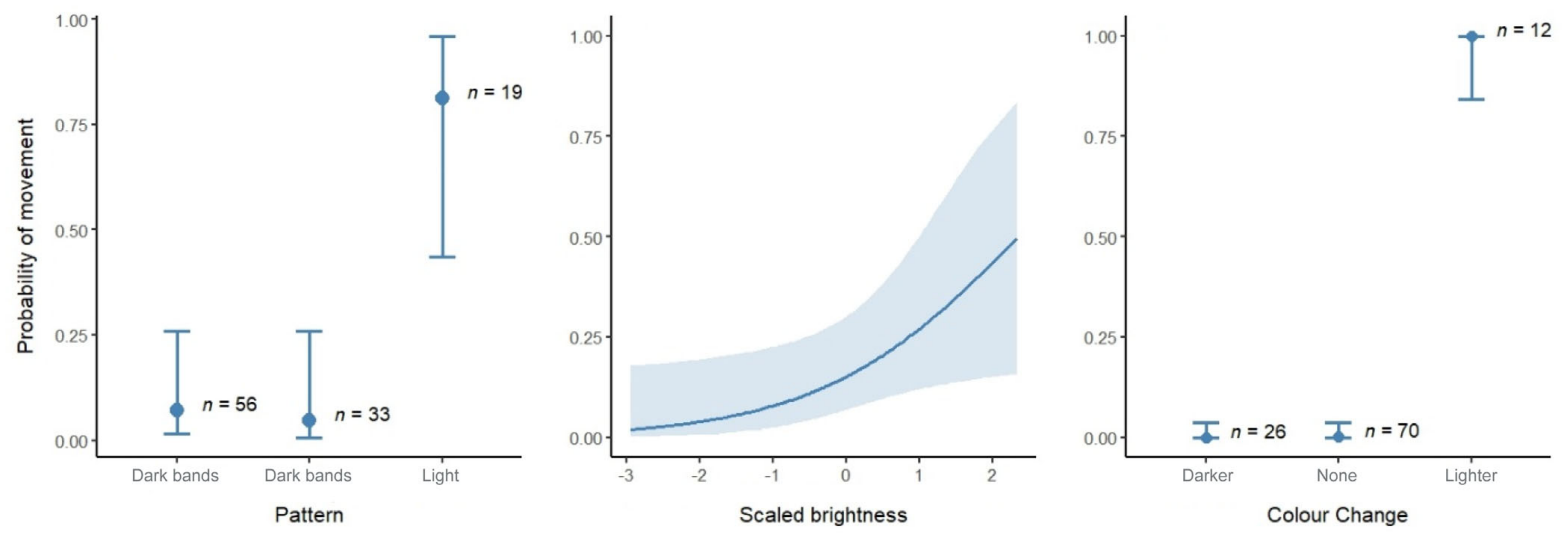
The impact of three measures of (a) colour pattern, (b) brightness and (c) colour change on the probability of tupong movement. Error bars and shading show 95% confidence intervals. Note that the pattern of error bars in (c) reflects the very strong relationship between colour change category and movement probability, with near‐perfect separation of outcomes. Fish moved in only one of 26 trials where they got darker, whilst in trials where fish got lighter, they remained still in only two of 12 cases.

Fish generally adopt colouration patterns that enable them to remain undetected to predators and prey (Smithers et al., 2018). Some fish species have evolved the capacity to undertake rapid colour changes to maintain camouflage even as the environmental or contextual situation itself changes (Leclercq et al., 2010). In this study, we found a clear link between fish pattern, brightness, colour change and movement behaviour, with those individuals that changed to be lighter moving significantly more than individuals that changed to be darker. The repeatability results indicate that individual tendencies also contributed, with some fish having a greater propensity to move or change colour more than others. We discuss several plausible mechanisms to describe our results, including the relationship between crypsis and behaviour, the dynamic environment surrounding the fish and stress‐induced colour change.

Animals regularly use colouration to avoid detection (crypsis) or deceive onlookers (mimicry). Camouflage is a type of crypsis where individuals use colour and pattern either to blend in with their background or to adopt high‐contrast disruptive colouration that breaks up their body outline. Mimicry, by contrast, involves individuals resembling another species or object to avoid detection (Stevens & Merilaita, 2009). Freshwater fish like tupong may have the ability to use either crypsis or mimicry to avoid detection by predators or prey. Tupong naturally live against a heterogeneous stream bed background. We found that tupong that became darker did not move and necessarily also produced a more complex colour pattern (stripes and bands). Such complex patterns could disrupt their body outline against a natural background (Drerup et al., 2024) and hence confuse a predator's detection system. Darker and more complex colouration may also assist fish in mimicking objects such as sticks, leaves and rocks on the stream bed to remain undetected (Sazima et al., 2006). While our results show clear links between movement, pattern, brightness and colour change, interpreting these differences as camouflage strategies requires consideration of predator perception. Future work could quantify tupong reflectance and contrast within the visual spectrum of their most common predators (Thetmeyer & Kils, 1995), which would allow testing of whether these colour patterns actually enhance concealment from predation.

Whilst it is plausible that those fish that became lighter did so to become better camouflaged against their white background, these were also the fish that moved. It is more likely that fish with a lighter and low‐contrast colour pattern may be more inconspicuous to predators while moving through a heterogeneous light environment (Attwell et al., 2021; Zylinski et al., 2009). This is in part due to caustics—the dynamic light patterns in the water caused by sunlight, ripples and debris on the water surface—which makes it harder for predators to detect lighter fish as they move (Cuthill et al., 2019; Matchette et al., 2018). Likewise, it may be much riskier for a fish to move when it has a dark colour pattern that is high contrast against a white background and thus conspicuous to predators (Endler, 1992; Szopa‐Comley et al., 2020). High‐contrast patterns or stripes can, in some cases, enhance camouflage during movement via the flicker‐fusion effect, whereby rapid movement exceeds the temporal resolution of a predator's visual system, causing patterns to blur and ultimately reducing detectability (Stevens, 2007; Umeton et al., 2017). However, this strategy is expected to be most effective at higher movement speeds and against predators with lower flicker‐fusion thresholds and less effective against avian predators with higher visual thresholds (Boström et al., 2016; Stevens, 2007). In the present study, where bird predation is likely to dominate and swimming speeds are generally slow, tupong may instead favour low‐contrast colouration during movement to remain inconspicuous.

Alternatively, it is plausible that movement itself is the key driver of colour change. If this is the case, then individuals may always be light during movement and always dark during stationary periods, regardless of background colour and pattern. Our repeatability analyses revealed moderate to high among‐individual differences in both movement and colour‐change behaviours. Collectively, this suggests that some fish are simply more active, more prone to colour‐change or potentially both. Individual behavioural tendencies may therefore interact with camouflage strategies, potentially reflecting aspects of personality (Kim & Velando, 2015). Interestingly, there was a marked difference in repeatability between individuals that consistently became lighter (*R* = 0.43) and those that became darker (*R* = 0.86), suggesting asymmetry in the expression of colour change. Because the best repeatability model did not include trial in the random effects structure, it is possible that the cumulative burden of capture and repeated testing influenced colour‐change expression. Under this scenario, some fish may have been more likely to express consistent darkening responses, potentially linked to stress or energy‐conservation priorities, whereas lightening, which may be more context‐dependent, was expressed more variably across trials. A future study assessing movement and colour change against different coloured and patterned backgrounds could determine whether movement or background drives tupong change colour and provide more insight into among‐individual variation in tupong behaviour.

Finally, hormones can play a crucial role in regulating colour in fish. The main hormones controlling pigmentation in fish, such as melanocyte‐stimulating hormones (e.g. *α*‐MSH), induce skin darkening by causing dispersion of dark pigment in cells (Green and Baker, 1991; Burton and Vokey, 2000). These hormones are also related to the stress responses of fishes (Sumpter et al., 1986). It is well known that individuals can vary in how stressful they find a given situation (Cockrem, 2013). Dark‐coloured tupong may have responded in this way because they had an elevated stress response following capture and sunlight stimulus, which also made them less likely to move. A similar relationship between an individual's colour change and stress hormone levels exists in Arctic char, where darker skin colour was observed in more subordinate fish (Höglund et al., 2000). However, in our study, aside from some fish remaining motionless, no other potentially stress‐related behaviours were observed throughout the experiment and movements appeared exploratory (i.e. not escape responses). This suggests that stress is unlikely to be regulating the colour change observed in our study.

Colour change plays a critical role in enabling fish to remain undetected by predators and prey. Here, we have shown that colour change is linked with movement behaviour in a freshwater fish, with lighter‐coloured fish moving more than darker‐coloured fish following a stimulus. We present several different pathways through which this interaction may materialise, but further research is required to test the exact mechanisms. It is clear, however, that colour and colour change have important consequences for behaviour. We suggest that the benefits of colour change are likely to be context dependent but may facilitate more efficient escape and predation, thus improving their short‐term performance and potentially long‐term fitness and survival.

## AUTHOR CONTRIBUTIONS

J.S.B.: Conceptualisation, data curation, formal analysis, investigation, funding acquisition, methodology, visualisation, writing–original draft, writing–review and editing. J.R.M.: Conceptualisation, formal analysis, resources, writing–review and editing.

## FUNDING INFORMATION

This research was funded by the Native Australian Animals Trust at the University of Melbourne.

## CONFLICT OF INTEREST STATEMENT

We declare that we have no competing interests.

## Supporting information


**TABLE S1.** AICc model comparisons for models with different colour measurements. Each colouration model was compared against the null model; the null model is shown only once, as its structure and results were identical across comparisons. Columns show the key colour predictor in each model, degrees of freedom, AICc and log‐likelihood. ‘Colour change’ indicates whether a fish became lighter, darker or showed no change, ‘pattern’ indicates whether a fish expressed dark bands, dark lines or a uniform light pattern, and ‘scaled brightness’ represents the standardised brightness score measured via pixel intensity.
**FIGURE S1.** Individual behavioural trial results. Rows correspond to individual fish (IDs shown on the left) and columns represent sequential trials 1, 2 and 3. Each tile shows whether a fish moved (text label ‘move’) or stayed (‘stay’) during that trial. Tile colour indicates brightness change: lighter (yellow), darker (orange) or no change (grey). Dark‐grey lines within each tile indicate the predominant colour pattern expressed: no line = light uniform pattern, horizontal line = dark lengthwise bands along the flanks, vertical bars = dark crosswise banding across the back.

## Data Availability

The data that support the findings of this study are openly available in Figshare at https://doi.org/10.26188/30093187.v1.

## References

[jfb70331-bib-0001] Attwell, J. R. , Ioannou, C. C. , Reid, C. R. , & Herbert‐Read, J. E. (2021). Fish avoid visually Noisy environments where prey targeting is reduced. The American Naturalist, 198(3), 421–432. 10.1086/715434 34403312

[jfb70331-bib-0002] Boström, J. E. , Dimitrova, M. , Canton, C. , Håstad, O. , Qvarnström, A. , & Ödeen, A. (2016). Ultra‐rapid vision in birds. PLoS One, 11(3), e0151099. 10.1371/journal.pone.0151099 26990087 PMC4798572

[jfb70331-bib-0003] Bray, D. J. , & Thompson, V. J. (2024). *Pseudaphritis urvillii* in Fishes of Australia. https://fishesofaustralia.net.au/home/species/403

[jfb70331-bib-0004] Bürkner, P.‐C. (2017). Brms: An R package for Bayesian multilevel models using Stan. Journal of Statistical Software, 80(1), 1–28. 10.18637/jss.v080.i01

[jfb70331-bib-0040] Burton, D. , & Vokey, J. E. (2000). The relative in vitro responsiveness of melanophores of winter flounder to α‐MSH and MCH. Journal of Fish Biology, 56(5), 1192–1200. 10.1111/j.1095-8649.2000.tb02133.x

[jfb70331-bib-0005] Chiao, C.‐C. , Chubb, C. , & Hanlon, R. T. (2015). A review of visual perception mechanisms that regulate rapid adaptive camouflage in cuttlefish. Journal of Comparative Physiology A, 201(9), 933–945. 10.1007/s00359-015-0988-5 25701389

[jfb70331-bib-0006] Cockrem, J. F. (2013). Individual variation in glucocorticoid stress responses in animals. General and Comparative Endocrinology, 181, 45–58. 10.1016/j.ygcen.2012.11.025 23298571

[jfb70331-bib-0007] Cooke, S. J. , Bergman, J. N. , Twardek, W. M. , Piczak, M. L. , Casselberry, G. A. , Lutek, K. , Dahlmo, L. S. , Birnie‐Gauvin, K. , Griffin, L. P. , Brownscombe, J. W. , Raby, G. D. , Standen, E. M. , Horodysky, A. Z. , Johnsen, S. , Danylchuk, A. J. , Furey, N. B. , Gallagher, A. J. , Lédée, E. J. I. , Midwood, J. D. , … Lennox, R. J. (2022). The movement ecology of fishes. Journal of Fish Biology, 101(4), 756–779. 10.1111/jfb.15153 35788929

[jfb70331-bib-0008] Cuthill, I. C. , Matchette, S. R. , & Scott‐Samuel, N. E. (2019). Camouflage in a dynamic world. Current Opinion in Behavioral Sciences, 30, 109–115. 10.1016/j.cobeha.2019.07.007

[jfb70331-bib-0009] Dickerson, A. L. , Rankin, K. J. , Cadena, V. , Endler, J. A. , & Stuart‐Fox, D. (2020). Rapid beard darkening predicts contest outcome, not copulation success, in bearded dragon lizards. Animal Behaviour, 170, 167–176. 10.1016/j.anbehav.2020.10.014

[jfb70331-bib-0010] Drerup, C. , Dunkley, K. , How, M. J. , & Herbert‐Read, J. E. (2024). Cuttlefish adopt disruptive camouflage under dynamic lighting. Current Biology, 34(14), e3255. 10.1016/j.cub.2024.06.015 38959882

[jfb70331-bib-0011] Duarte, R. C. , Flores, A. A. V. , & Stevens, M. (2017). Camouflage through colour change: Mechanisms, adaptive value and ecological significance. Philosophical Transactions of the Royal Society, B: Biological Sciences, 372(1724), 20160342. 10.1098/rstb.2016.0342 PMC5444063 28533459

[jfb70331-bib-0012] Duarte, R. C. , Wade, N. M. , & Stevens, M. (2025). Animal colour change: Proximate mechanisms, evolutionary ecology and response to anthropogenic impacts. Journal of Experimental Biology, 228(11), jeb249764. 10.1242/jeb.249764 40497776

[jfb70331-bib-0013] Endler, J. A. (1992). Signals, signal conditions, and the direction of evolution. The American Naturalist, 139, S125–S153. 10.1086/285308

[jfb70331-bib-0014] Figon, F. , & Casas, J. (2018). Morphological and physiological colour changes in the animal kingdom. In Encyclopedia of Life Sciences (pp. 1–11). John Wiley & Sons, Ltd.

[jfb70331-bib-0015] Fujii, R. (2000). The regulation of motile activity in fish chromatophores. Pigment Cell Research, 13(5), 300–319. 10.1034/j.1600-0749.2000.130502.x 11041206

[jfb70331-bib-0041] Furey, N. B. , Hinch, S. G. , Bass, A. L. , Middleton, C. T. , Minke‐Martin, V. , & Lotto, A. G. (2016). Predator swamping reduces predation risk during nocturnal migration of juvenile salmon in a high‐mortality landscape. Journal of Animal Ecology, 85(4), 948–959. 10.1111/1365-2656.12528 27159553

[jfb70331-bib-0039] Green, J. A. , & Baker, B. I. (1991). The influence of repeated stress on the release of melanin‐concentrating hormone in the rainbow trout. Journal of Endocrinology, 128(2), 261–266. 10.1677/joe.0.1280261 2005416

[jfb70331-bib-0016] Green, S. D. , Duarte, R. C. , Kellett, E. , Alagaratnam, N. , & Stevens, M. (2019). Colour change and behavioural choice facilitate chameleon prawn camouflage against different seaweed backgrounds. Communications Biology, 2(1), 230. 10.1038/s42003-019-0465-8 31263774 PMC6588621

[jfb70331-bib-0017] Höglund, E. , Balm, P. H. M. , & winberg, S. (2000). Skin darkening, a potential social signal in subordinate Arctic charr (*Salvelinus alpinus*): The regulatory role of brain monoamines and pro‐opiomelanocortin‐derived peptides. Journal of Experimental Biology, 203(11), 1711–1721. 10.1242/jeb.203.11.1711 10804161

[jfb70331-bib-0018] Kim, S.‐Y. , & Velando, A. (2015). Phenotypic integration between antipredator behavior and camouflage pattern in juvenile sticklebacks. Evolution, 69(3), 830–838. 10.1111/evo.12600 25572122

[jfb70331-bib-0019] Leclercq, E. , Taylor, J. F. , & Migaud, H. (2010). Morphological skin colour changes in teleosts. Fish and Fisheries, 11(2), 159–193. 10.1111/j.1467-2979.2009.00346.x

[jfb70331-bib-0020] Ligon, R. A. , & McGraw, K. J. (2013). Chameleons communicate with complex colour changes during contests: Different body regions convey different information. Biology Letters, 9(6), 20130892. 10.1098/rsbl.2013.0892 24335271 PMC3871380

[jfb70331-bib-0021] Matchette, S. R. , Cuthill, I. C. , & Scott‐Samuel, N. E. (2018). Concealment in a dynamic world: Dappled light and caustics mask movement. Animal Behaviour, 143, 51–57. 10.1016/j.anbehav.2018.07.003

[jfb70331-bib-0022] Morrongiello, J. R. , Bond, N. R. , Crook, D. A. , & Wong, B. B. M. (2010). Nuptial coloration varies with ambient light environment in a freshwater fish. Journal of Evolutionary Biology, 23(12), 2718–2725. 10.1111/j.1420-9101.2010.02149.x 20964785

[jfb70331-bib-0023] R Core Team . (2024). R: A Language and Environment for Statistical Computing, R Foundation for Statistical Computing. R Foundation for Statistical Computing.

[jfb70331-bib-0024] Sazima, I. , Carvalho, L. N. , Mendonça, F. P. , & Zuanon, J. (2006). Fallen leaves on the water‐bed: Diurnal camouflage of three night active fish species in an Amazonian streamlet. Neotropical Ichthyology, 4, 119–122.

[jfb70331-bib-0025] Schindelin, J. , Arganda‐Carreras, I. , Frise, E. , Kaynig, V. , Longair, M. , Pietzsch, T. , Preibisch, S. , Rueden, C. , Saalfeld, S. , Schmid, B. , Tinevez, J.‐Y. , White, D. J. , Hartenstein, V. , Eliceiri, K. , Tomancak, P. , & Cardona, A. (2012). Fiji: An open‐source platform for biological‐image analysis. Nature Methods, 9(7), 676–682. 10.1038/nmeth.2019 22743772 PMC3855844

[jfb70331-bib-0026] Smithers, S. P. , Rooney, R. , Wilson, A. , & Stevens, M. (2018). Rock pool fish use a combination of colour change and substrate choice to improve camouflage. Animal Behaviour, 144, 53–65. 10.1016/j.anbehav.2018.08.004

[jfb70331-bib-0027] Stevens, M. (2007). Predator perception and the interrelation between different forms of protective coloration. Proceedings of the Royal Society B: Biological Sciences, 274(1617), 1457–1464. 10.1098/rspb.2007.0220 PMC1950298 17426012

[jfb70331-bib-0028] Stevens, M. , & Merilaita, S. (2009). Animal camouflage: Current issues and new perspectives. Philosophical Transactions of the Royal Society, B: Biological Sciences, 364(1516), 423–427. 10.1098/rstb.2008.0217 PMC2674078 18990674

[jfb70331-bib-0029] Stuart‐Fox, D. , & Moussalli, A. (2011). Camouflage in colour‐changing animals: Trade‐offs and constraints. In M. Stevens & S. Merilaita (Eds.), Animal camouflage: Mechanisms and function (pp. 237–253). Cambridge.

[jfb70331-bib-0030] Sugimoto, M. (2002). Morphological color changes in fish: Regulation of pigment cell density and morphology. Microscopy Research and Technique, 58(6), 496–503. 10.1002/jemt.10168 12242707

[jfb70331-bib-0031] Sumpter, J. P. , Dye, H. M. , & Benfey, T. J. (1986). The effects of stress on plasma ACTH, *α*‐MSH, and cortisol levels in salmonid fishes. General and Comparative Endocrinology, 62(3), 377–385. 10.1016/0016-6480(86)90047-X 3021561

[jfb70331-bib-0032] Szopa‐Comley, A. W. , Donald, W. G. , & Ioannou, C. C. (2020). Predator personality and prey detection: Inter‐individual variation in responses to cryptic and conspicuous prey. Behavioral Ecology and Sociobiology, 74(6), 70. 10.1007/s00265-020-02854-9

[jfb70331-bib-0033] Thetmeyer, H. , & Kils, U. (1995). To see and not be seen: The visibility of predator and prey with respect to feeding behaviour. Marine Ecology Progress Series, 126, 1–8.

[jfb70331-bib-0034] Umbers, K. D. L. , Fabricant, S. A. , Gawryszewski, F. M. , Seago, A. E. , & Herberstein, M. E. (2014). Reversible colour change in Arthropoda. Biological Reviews, 89(4), 820–848. 10.1111/brv.12079 24495279

[jfb70331-bib-0035] Umeton, D. , Read, J. C. A. , & Rowe, C. (2017). Unravelling the illusion of flicker fusion. Biology Letters, 13(2), 20160831. 10.1098/rsbl.2016.0831 28148834 PMC5326512

[jfb70331-bib-0036] Whitaker, K. W. , Alvarez, M. , Preuss, T. , Cummings, M. E. , & Hofmann, H. A. (2021). Courting danger: Socially dominant fish adjust their escape behavior and compensate for increased conspicuousness to avian predators. Hydrobiologia, 848(16), 3667–3681. 10.1007/s10750-020-04475-9

[jfb70331-bib-0037] Zimova, M. , Hackländer, K. , Good, J. M. , Melo‐Ferreira, J. , Alves, P. C. , & Mills, L. S. (2018). Function and underlying mechanisms of seasonal colour moulting in mammals and birds: What keeps them changing in a warming world? Biological Reviews, 93(3), 1478–1498. 10.1111/brv.12405 29504224

[jfb70331-bib-0038] Zylinski, S. , Osorio, D. , & Shohet, A. J. (2009). Cuttlefish camouflage: Context‐dependent body pattern use during motion. Proceedings of the Royal Society B: Biological Sciences, 276(1675), 3963–3969. 10.1098/rspb.2009.1083 PMC2825777 19692411

