# Supplementary material for: Dynamic colour change predicts movement behaviour in a diadromous fish
Source: J Fish Biol. 2026 Feb 19;108(6):2205–10. doi: 10.1111/jfb.70331 (PMC13357239; doi:10.1111/jfb.70331)
Supplement: Supplementary file 1 — TABLE S1. AICc model comparisons for models with different colour measurements. Each colouration model was compared against the null model; the null model is shown only once, as its structure and results were identical across comparisons. Columns show the key colour predictor in each model, degrees of freedom, AICc and log‐likelihood. ‘Colour change’ indicates whether a fish became lighter, darker or showed no change, ‘pattern’ indicates whether a fish expressed dark bands, dark lines or a uniform light pattern, and ‘scaled brightness’ represents the standardised brightness score measured via pixel intensity. FIGURE S1. Individual behavioural trial results. Rows correspond to individual fish (IDs shown on the left) and columns represent sequential trials 1, 2 and 3. Each tile shows whether a fish moved (text label ‘move’) or stayed (‘stay’) during that trial. Tile colour indicates brightness change: lighter (yellow), darker (orange) or no change (grey). Dark‐grey lines within each tile indicate the predominant colour pattern expressed: no line = light uniform pattern, horizontal line = dark lengthwise bands along the flanks, vertical bars = dark crosswise banding across the back. [file JFB-108-2205-s001.docx]

**Supplementary materials**

Supp. Table 1. AICc model comparisons for models with different colour measurements. Each colouration model was compared against the null model; the null model is shown only once, as its structure and results were identical across comparisons. Columns show the key colour predictor in each model, degrees of freedom, AICc, and log-likelihood. ‘Colour change’ indicates whether a fish became lighter, darker, or showed no change; ‘Pattern’ indicates whether a fish expressed dark bands, dark lines, or a uniform light pattern; and ‘Scaled brightness’ represents the standardised brightness score measured via pixel intensity.

| Model | df | AICc | LL |
| --- | --- | --- | --- |
| Colour change | 4 | 82.19 | -36.90 |
| Pattern | 4 | 90.17 | -40.89 |
| Scaled brightness | 3 | 108.70 | -51.23 |
| null | 2 | 111.85 | -53.87 |


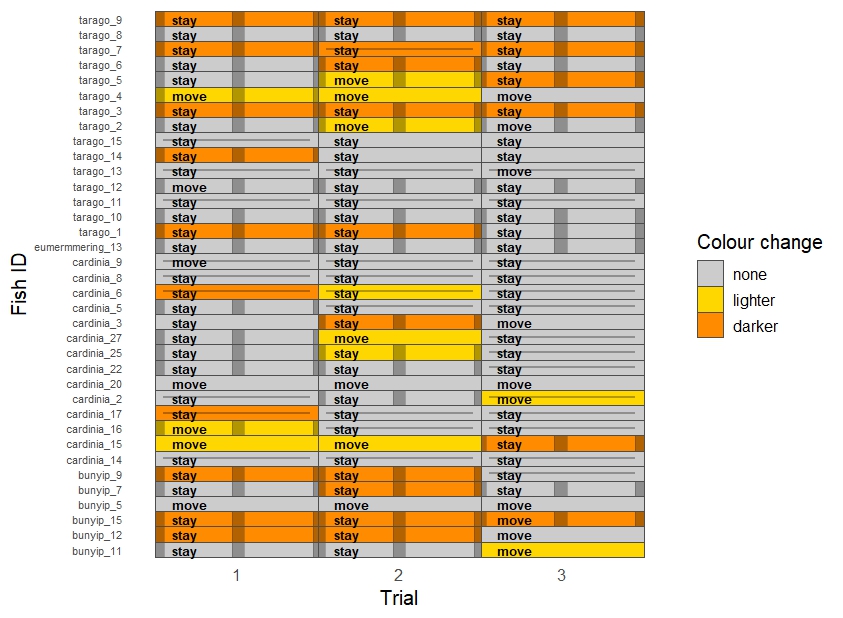


Supp. Figure 1. Individual behavioural trial results. Rows correspond to individual fish (IDs shown on the left), and columns represent sequential trials 1, 2, and 3. Each tile shows whether a fish moved (text label “move”) or stayed (“stay”) during that trial. Tile colour indicates brightness change: lighter (yellow), darker (orange), or no change (grey). Dark grey lines within each tile indicate the predominant colour pattern expressed: no line = light uniform pattern, horizontal line = dark lengthwise bands along the flanks, vertical bars = dark crosswise banding across the back.
